# Self-harm incidence among children and young people 2019–2023: time series analysis of electronic health records in Greater Manchester, England

**DOI:** 10.1136/bmjment-2025-301615

**Published:** 2025-06-09

**Authors:** Louise Jane Hussey, Evangelos Kontopantelis, Nav Kapur, Richard Williams, Pearl Mok, Darren M Ashcroft, Shruti Garg, Carolyn Chew Graham, Karina Lovell, Roger Thomas Webb

**Affiliations:** 1 Division of Psychology & Mental Health, The University of Manchester, Manchester Academic Health Science Centre (MAHSC), Manchester, UK; 2 National Institute for Health and Care Research (NIHR) Applied Research Collaboration - Greater Manchester (ARC-GM), The University of Manchester, Manchester, UK; 3 National Institute for Health and Care Research (NIHR) Manchester Biomedical Research Centre, The University of Manchester, Manchester, UK; 4 Division of Informatics, Imaging & Data Sciences, The University of Manchester, Manchester Academic Health Science Centre (MAHSC), Manchester, UK; 5 Mersey Care NHS Foundation Trust, Liverpool, England, UK; 6 National Institute for Health and Care Research (NIHR) Greater Manchester Patient Safety Research Collaboration, The University of Manchester, Manchester, UK; 7 National Confidential Inquiry into Suicide and Safety in Mental Health (NCISH), The University of Manchester, Manchester, UK; 8 Division of Pharmacy & Optometry, The University of Manchester, Manchester Academic Health Science Centre (MAHSC), Manchester, UK; 9 Royal Manchester Children’s Hospital, Manchester University NHS Foundation Trust, Manchester, UK; 10 School of Medicine, Faculty of Medicine and Health Sciences, Keele University, Keele, UK; 11 Mental Health Nursing Research Unit, Greater Manchester Mental Health NHS Foundation Trust, Manchester, UK; 12 Division of Nursing, Midwifery & Social Work, University of Manchester, Manchester Academic Health Science Centre (MAHSC), Manchester, UK

**Keywords:** COVID-19, Child & adolescent psychiatry, Suicide & self-harm, Anxiety disorders, Depression & mood disorders

## Abstract

**Background:**

The mental health of children and adolescents has declined in recent years. Self-harm is frequently an expression of this psychological distress.

**Objectives:**

To examine trends in self-harm incidence among 10–24-year olds between January 2019–December 2023.

**Methods:**

We conducted time-series analyses of all incident episodes of self-harm among 10–24-year olds using the Greater Manchester Care Record. The observation period was split into four phases: pre-pandemic (1/2019–2/2020); pandemic phase 1 (3/2020–6/2021); pandemic phase 2 (7/2021–12/2022) and post-pandemic (1/2023–12/2023). Rate ratios by sex, age, ethnicity and Indices of Multiple Deprivation were modelled using negative binomial regression.

**Findings:**

Self-harm incidence rates decreased significantly in the post-pandemic phase, compared with the pre-pandemic period (male—incident rate ratios (IRR) 0.72; 95% CI 0.62 to 0.84, female IRR 0.85; 95% CI 0.74 to 0.99). In females, this followed increased rates, rising by 18% in pandemic phase 2 (IRR 1.18; 95% CI 1.04 to 1.34). In males, rates decreased throughout the study period. Incidence rates were lowest for 10–12 year olds. However, the greatest increase was observed in this age group, with rates in pandemic phase 2 being almost two times that seen pre-pandemic for females (IRR 1.91; 95% CI 1.47 to 2.48). The change in rates among females was also most marked in the least deprived neighbourhoods, rising by more than 50% (IRR 1.54; 95% CI 1.21 to 1.95) in pandemic phase 2.

**Conclusions:**

Our results indicate a decrease in self-harm incidence during 2023. Analysis by age group showed the greatest increase in rates in 10–12-year olds. Further research is needed to confirm these findings and to identify the mechanisms driving these trends.

**WHAT IS ALREADY KNOWN ON THIS TOPIC**

- The mental health of children and young people (CYP) has declined in recent years and self-harm is frequently an expression of this psychological distress with prevalence highest in females and individuals living in more deprived neighbourhoods.
- The COVID-19 pandemic and the associated societal restriction measures that started in March 2020 have been shown to have a negative impact on the mental well-being of children and adolescents.

**WHAT THIS STUDY ADDS**

- Self-harm incidence rates showed a significant decrease during the study’s post-pandemic observation period (January–December 2023).
- The greatest increase in age-specific rates during the pandemic was among 10–12-year olds.

**HOW THIS STUDY MIGHT AFFECT RESEARCH, PRACTICE OR POLICY**

- Increased incidence rates among young teenage girls may suggest younger CYP are increasingly being exposed to risk factors for harmful behaviours. It is essential to identify risks of self-harm and barriers to care for different sectors of society and to enable appropriate and timely care for mental health problems in all CYP.

## Background

 The mental health of children and young people (CYP) has declined in recent years.^1^ A survey of 2370 CYP in England found that in 2023, 20.3% of 8–16-year olds had a probable mental illness compared with 12.5% in 2017; the reported rise was even greater among 17–19-year olds: 23.3% versus 10.1%.^2^ Self-harm, though not a diagnosable mental illness, is frequently an expression of psychological distress.^3^ It is typically first reported during adolescence and is one of the most important predictors of subsequent suicidal behaviour.^4^ Studies have shown that self-harm occurs more frequently among people of lower socioeconomic status,^5^ but less evidence regarding self-harm risk and ethnicity has been generated to date. However, one study examining presentations in emergency departments (EDs) in three English cities found rates were highest in white CYP compared with black, South Asian and other non-white groups. Between 2009 and 2016 increased rates were observed in all ethnic groups examined, but increases were slightly greater in people from ethnic minorities compared with those of white ethnicity. CYP from non-white groups were also less likely to receive a specialist psychosocial assessment.^6^ A UK-based cohort study examining data from over 11 000 14-year olds estimated the prevalence at 15.4% with a female to male ratio of 2:6. Greater likelihood of self-harm in girls was associated with not confiding in family members and frequent social media use. In boys, associations were found with bullying and same-sex attraction.^7^ Other studies have shown the negative effect of social media usage, particularly for adolescents during a significant period of mental development making them more vulnerable.^8^

The COVID-19 pandemic and the associated societal restrictions that started in March 2020 have been shown to have a negative impact on the mental health of CYP.^9^ School closures and social isolation might have resulted in an increased incidence of self-harm among some CYP, complicated by negative coping strategies and increased rumination and overthinking.^10^ Greater stringency of lockdown measures was found to be associated with increased self-harm presentation frequencies.^10^ Studies based on electronic health records have examined the impact of the COVID-19 pandemic on self-harm rates in CYP.^11^ These have shown an initial decrease in self-harm episodes during the early stage of the pandemic possibly due to changes in the delivery of primary care services and help-seeking behaviour but then rates subsequently increased resulting in higher levels than those seen pre-pandemic.

Greater Manchester (GM) is a large conurbation in the North-West region of England, UK. Compared with the entire population of England, GM’s population of approximately 2.8 million people has relatively high levels of deprivation and is more ethnically diverse.^12^ During the UK Government’s response to the COVID-19 pandemic, there were various stages of mandatory ‘lockdowns’ and other societal restrictions, with GM residents experiencing some of the most stringent virus containment measures over lengthier time periods.

### Objectives

The aim of this study was to use electronic health records across GM to examine how self-harm incidence rates in CYP changed during 5 years from 2019 through 2023. This observation period encompasses a year pre-COVID-19, the acute phase of the pandemic and 30 months since societal restrictions were lifted. We also aimed to assess whether these outcome frequencies varied by sex, age, ethnicity and neighbourhood-level Index of Multiple Deprivation (IMD).

## Methods

We conducted a time-series analysis using incident episodes of self-harm recorded in the GM Care Record (GMCR).^13^ This database, established in 2019, holds approximately three million patient records from around 450 general practices across the whole GM conurbation. A research protocol was approved enabling access to data pertaining to individual patients in accordance with the National Control of Patient Information notice.^14^ We extracted monthly totals of all clinical codes, indicating incident self-harm episodes recorded among patients aged 10–24 years over a 5-year study period, from 1 January 2019 to 31 December 2023. All previous entries in a patient’s electronic health record were examined to ensure that episode counts were the first reports of self-harm and therefore incident episodes. The incident counts were stratified by individual-level sex, age group (10–12, 13–16, 17–29, 20–24 years), ethnicity (white, black and Asian) and neighbourhood-level deprivation as measured by IMD 2019 quintiles. Some ethnicity and IMD data were missing (online supplemental table S1). However, all incident self-harm episodes were included in the analyses by sex and age group. Incidence rates were calculated using monthly estimates of population denominators extracted from three data sources: GMCR Census 2021; (used to validate the proportional distribution by sex, age, ethnicity and IMD)^15^ and NHS Digital GP practice list information to provide monthly population size data.^16^

To enable examination of changes in incidence rates throughout the observation period, we divided the 60-month dataset into four time periods. The three epochs since the start of the pandemic (from March 2020) were compared with the preceding pre-pandemic period as follows:

- Pre-pandemic: January 2019–February 2020 (months 1–14).
- Pandemic phase 1 (societal restrictions in place): March 2020–June 2021 (months 15–30).
- Pandemic phase 2 (all restrictions lifted): July 2021–December 2022 (months 31–48).
- Post-pandemic: January–December 2023 (months 49–60).

The fourth phase was labelled as ‘post-pandemic’, although we acknowledge that the SARS-CoV-2 virus continued to be transmitted endemically within the population afterwards. In the UK, the Winter of 2022 was the last time when the COVID-19 vaccination booster was offered to individuals under the age of 75 (without clinical vulnerabilities or due to professional role).^17^ As such, we considered it reasonable to classify this last 12 months (2023) of the observation period as a separate ‘post-pandemic’ phase.

The numbers of incident self-harm episodes were aggregated to give monthly counts. These counts were then stratified according to the four pandemic time periods as described previously. Monthly incident rates per 100 000 person-months and incident rate ratios (IRR) (and their 95% CIs) were calculated using negative binomial regression by sex, age group, ethnicity and IMD quintile. The model, in addition to these covariates, included a time variable (month 1 to 60), a pandemic phase variable (1 to 4) and was adjusted for seasonality by including a variable for calendar month. IRRs were estimated to compare rates during pandemic phase 1, pandemic phase 2 and the post-pandemic phase with the pre-pandemic period as the reference category. Model outputs were obtained using the ‘margins’ command. Analyses were performed in Stata V17.

Results of these analyses were presented in two plots for each sociodemographic subgroup: one to illustrate the incidence rate per 100 000 person-months and thereby depicting temporal changes in these rates and another to show the IRRs comparing incidence at the different pandemic phases to the pre-pandemic period (the reference category, IRR=1).

In addition to this examination of the self-harm incidence rates, we also carried out similar analyses for the total number of recorded self-harm episodes (ie, incident plus repeat episodes) per month. These additional models informed our interpretation of the primary results, specifically to discern whether the observed trends were being driven by CYP self-harming for the first time or by changes in the episodic rate of individuals already recorded as having self-harmed. Further secondary analyses (by sex only) estimated the incidence and prevalence rates of common mental disorders (anxiety disorders and depression) and also the antidepressant prescribing rate. We aimed to thereby gain a greater understanding of how temporal trends in self-harm were related to the wider picture of mental distress in CYP and the unique challenges presented during this 5-year period. All secondary analyses are presented in the supplementary materials.

### Patient and public involvement

There was no patient or public involvement in this study.

## Findings

### Incidence and prescribing rates across the whole study period

**Figure 1:**
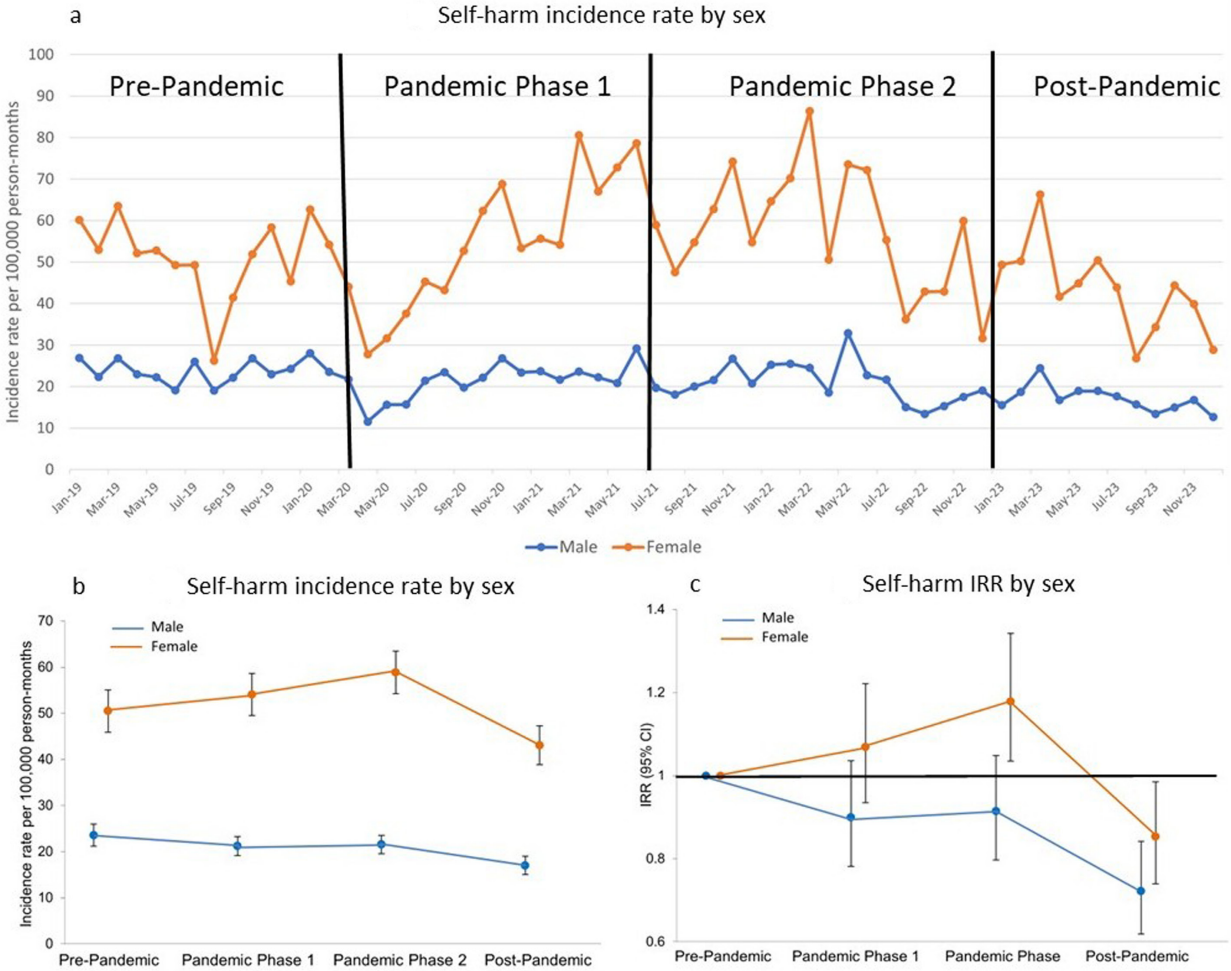
Temporal trends in self-harm incidence by sex. (a) Sex-specific self-harm incidence rates per 100 000 person-months and (b and c) incidence rates per 100 000 person-months (95% CI) and incidence rate ratios (IRR) by sex (vs the pre-pandemic period, 95% CI).

Over the 5-year study period, the dataset extracted from the GMCR held 13 033 incident self-harm episodes (online supplemental table S1). The incidence rate (per 100 000 person-months) in females was two times that observed in males (IRR 2.14; 95% CI 1.87 to 2.45) (figure 1). For both males and females, incidence rates were lowest in 10–12-year olds. In males, rates were highest in 17–19-year olds and were over four times higher (IRR 4.54; 95% CI 3.39 to 6.07) than the youngest age group. In females, the highest rates were observed in the 13–16-year olds; seven times higher than those in the 10–12 age group (IRR 7.22; 95% CI 5.66 to 9.22). Incidence rates were between two and four times lower for individuals of black (male IRR 0.23; 95% CI 0.13 to 0.38, female IRR 0.36; 95% CI 0.27 to 0.49) and Asian (male IRR 0.32; 95% CI 0.23 to 0.42, female IRR 0.33; 95% CI 0.27 to 0.41) ethnicity when compared with the white ethnic group. Overall, self-harm incidence rates were lower with decreasing levels of neighbourhood deprivation. Rates in the least deprived quintile (IMD5) were approximately 40%–50% lower than those in the most deprived quintile (IMD1) (male IRR 0.48; 95% CI 0.36 to 0.64, female IRR 0.60; 95% CI 0.49 to 0.74).

### Temporal trends by sex and age

**Figure 2:**
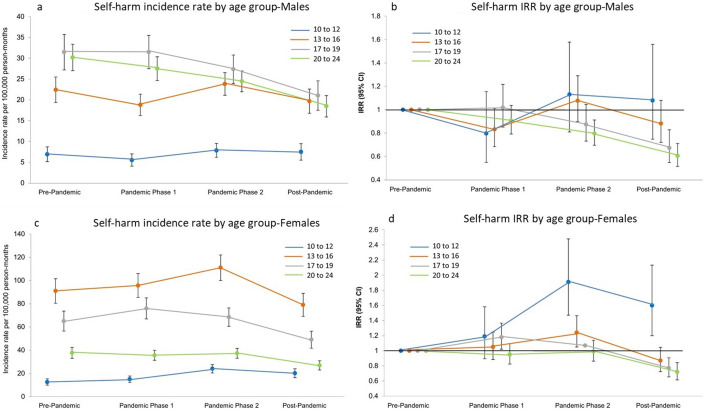
Temporal trends in self-harm incidence by age group. (a and c) Incidence rates per 100 000 person-months (95% CI) and (b and d) incidence rate ratios (IRR) by sex (vs the pre-pandemic period, 95% CI).

Self-harm incidence rates decreased in the early stages of the pandemic and the accompanying imposition of societal restrictions and the closure of educational establishments in March 2020 (figure 1a). Rates in males then increased, and then a slight decrease was observed throughout the rest of pandemic phases 1 and 2. Schools reopened in September 2020, which (as illustrated in figure 1a) seems to coincide with the incidence in females, increasing beyond that observed prior to societal restrictions being imposed. Incidence in females then continued to increase during both pandemic phases, with the rates in pandemic phase 2 being 18% (IRR 1.18; 95% CI 1.04 to 1.34) higher compared with that seen pre-pandemic (figure 1c and online supplemental table S2). During 2023 (the post-pandemic period in our study), a significant decrease was observed, with rates falling below those in the pre-pandemic period (in male IRR 0.72; 95% CI 0.62 to 0.84, and in female IRR 0.85; 95% CI 0.74 to 0.99). The graphs in figure 2 illustrate how self-harm incidence rates changed over the 60-month observation period by age group. As outlined previously, the 10–12 age group had the lowest rates. However, as illustrated in the IRR plots and online supplemental table S2, rates in this younger group have shown the greatest relative increases compared with rates observed pre-pandemic, for both males and females, particularly during pandemic phase 2. In females, the incidence rate was almost two times that of the pre-pandemic rate (IRR 1.91; 95% CI 1.47 to 2.48). A decrease in incidence rates between pandemic phase 2 and the post-pandemic period was observed for all age groups, resulting in the rates of the two older groups being significantly lower than the pre-pandemic rates (in males 17–19 years: IRR 0.67; 95% CI 0.55 to 0.83, 20–24 years IRR 0.61; 95% CI 0.52 to 0.71, and in females 17–19 years: IRR 0.77; 95% CI 0.65 to 0.90; 20–24 years IRR 0.72; 95% CI 0.61 to 0.84).

Rates for all self-harm episodes (as opposed to incidence rates) are illustrated in the supplementary materials (online supplemental figures S1–S4). In general, for episode rates, the patterns between demographic subgroups were comparable to those observed for incidence rates apart from one notable exception; in females, the rate in the 17–19 years age group was observed to be similar to those aged 13–16 years, with both age groups having episode rates approximately 11 times higher than the youngest age group (online supplemental figure S2).

To explore whether this observed fall in incidence rates from pandemic phase 2 to the post-pandemic period was specific to self-harm, we conducted similar analysis examining incident diagnoses and prevalence (episodes) of common mental disorders (anxiety disorder and depression) (online supplemental figures S5 and S6) and antidepressant prescribing rates (online supplemental figure S7). In males, self-harm incidence rates showed a small decrease in pandemic phase 1. For common mental disorders, incidence rates decreased significantly in the early stages of the pandemic for both malesand females and remained lower than those observed pre-pandemic for the whole of our observation period. Following an increase in pandemic phase 2, rates also decreased in the post-pandemic period. A similar trend is observed in the analysis of common mental disorder prevalence (episode rates). However, this fall in incidence was small when compared with the much greater fall observed in self-harm rates. The rate of antidepressant prescribing increased throughout the first 4 years of our study period and remained at a similar level in 2023.

### Temporal trends by ethnicity and neighbourhood deprivation quintiles

**Figure 3:**
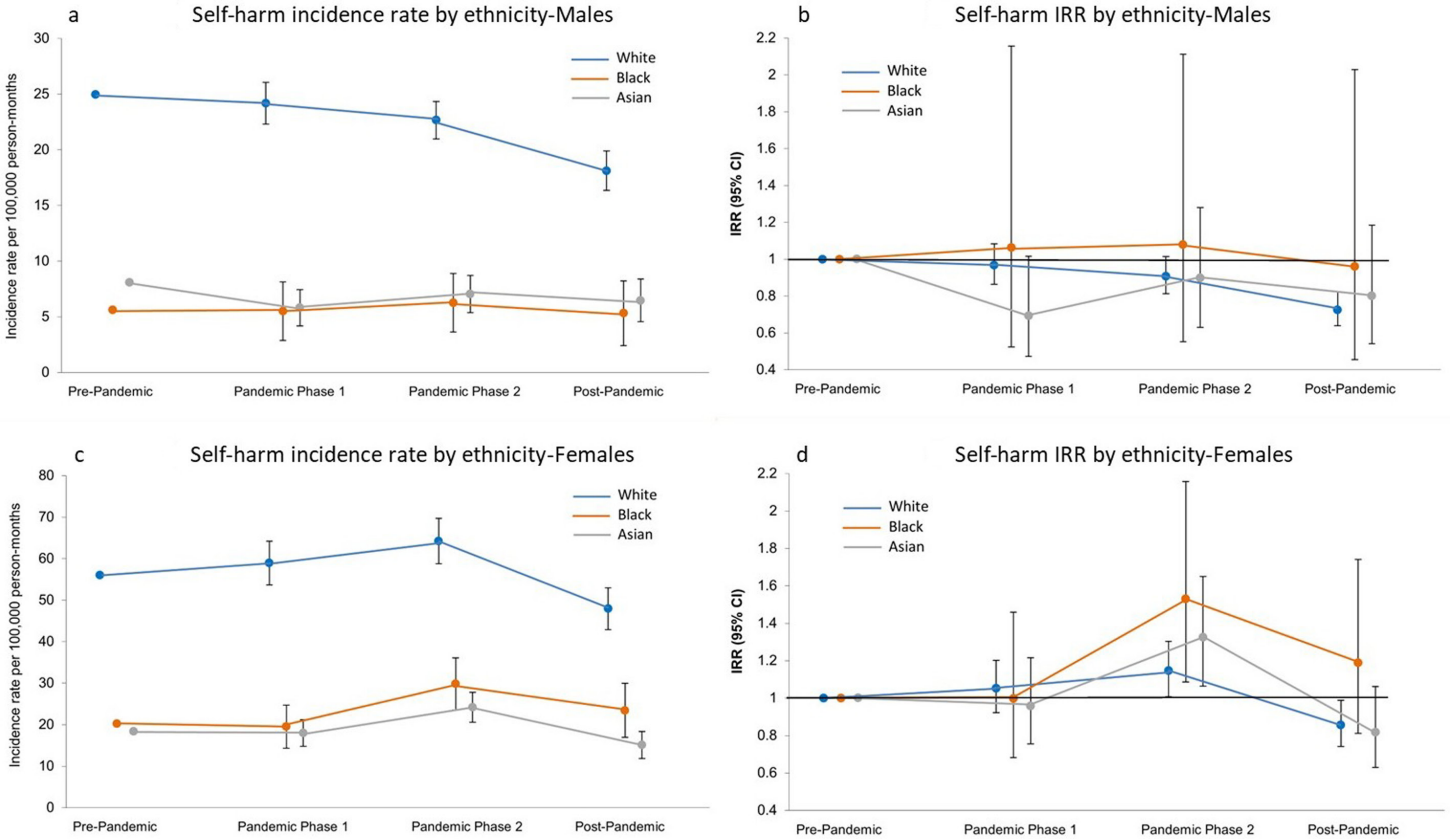
Temporal trends in self-harm incidence by ethnicity. (a and c) Incidence rates per 100 000 person-months (95% CI) and (b and d) incidence rate ratios (IRR) by sex (vs the pre-pandemic period, 95% CI).

**Figure 4:**
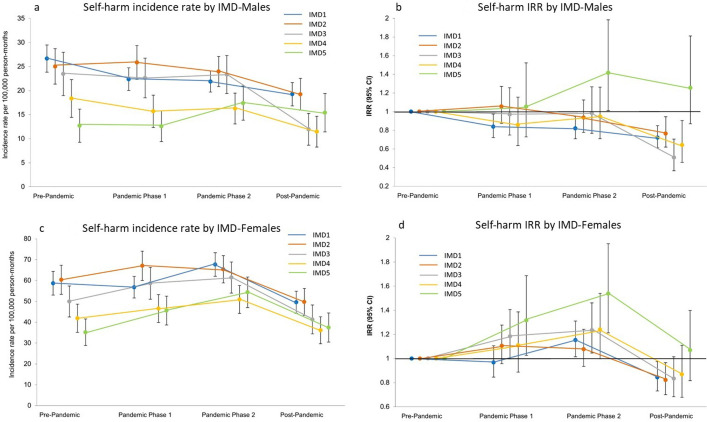
Temporal trends in self-harm incidence by neighbourhood deprivation quintile.* (a and c) Incidence rates per 100 000 person-months (95% CI) and (b and d) incidence rate ratios (IRR) by sex (vs the pre-pandemic period, 95% CI). *Neighbourhood-level Index of Multiple Deprivation (IMD): IMD1, most deprived; IMD5, least deprived.

Analysis by ethnic group and neighbourhood deprivation quintile also showed a decrease in self-harm incidence rates in all demographic subgroups examined during the last year of the study’s observation period. However, this decrease in incidence rates compared with the pre-pandemic period was only shown to be significant in the white ethnic group (in male IRR 0.73; 95% CI 0.64 to 0.82, and in female IRR 0.86; 95% CI 0.74 to 0.99) (figure 3 and online supplemental table S3). Smaller event counts have resulted in wide CIs for other ethnic groups, particularly for those of black ethnicity. However, our results suggest that for females, in pandemic phase 2, there was a greater increase rates in those of black and Asian ethnicities than there was for the white ethnic group (white IRR 1.15; 95% CI 1.01 to 1.30, black IRR 1.53; 95% CI 1.09 to 2.16, Asian IRR 1.32; 95% CI 1.06 to 1.65). As illustrated in figure 4 and online supplemental table S3, self-harm incidence rates in females generally increased for all deprivation quintiles throughout pandemic phases 1 and 2, prior to the post-pandemic decrease. The change in incidence rates was most notable in the least deprived quintile (IMD5). Rates in this group were lowest during the pre-pandemic period but showed the greatest increase rising by 54% (IRR 1.54; 95% CI 1.21 to 1.95) in pandemic phase 2. There was a different trend in males with a decrease in incidence observed throughout the study period, apart from the least deprived quintile, which rose by 42% (IRR 1.42; 95% CI 1.01 to 1.98). As with all other deprivation quintiles, for both males and females, rates for the least deprived quintile then decreased in the post-pandemic period. However, they remained higher than the rates observed at the start of our study period.

## Discussion

### Statement of principal findings

Self-harm incidence rates showed a significant decrease in the last 12 months of the study’s observation period (January–December 2023), compared with rates in the pre-pandemic period. In males, a greater fall occurred after a gradual decrease throughout the pre-pandemic and pandemic phases. However, in females, this decrease during the last 12 months followed increased rates in pandemic phases 1 and 2. The incidence rate in 13–16-year old females was higher than for any other age group. The youngest age group (10–12 years) had the lowest rates but showed the greatest percentage increase in incidence, particularly in pandemic phase 2. The change in incidence rates was most notable in the least deprived quintile (IMD5); they were lowest during the pre-pandemic period but showed the greatest percentage increases during pandemic phases 1 and 2. As with all other deprivation quintiles, rates then decreased during the post-pandemic period, although they remained higher than the rates seen at the start of the observation period.

### Strengths and limitations of this study

Utilisation of GMCR data enabled examination of temporal trends in self-harm rates across the whole GM population over a relatively recent 5-year period through to the end of the 2023 calendar year, stratified by individual-level ethnicity and neighbourhood-level deprivation. Comparable studies including this recent timeframe were not evident in the published literature at the time of writing. The GM conurbation is relatively ethnically diverse and has higher levels of deprivation than the national average. As such, this may have an impact on the external validity of the study when comparing our results with other areas nationally. In addition, GM experienced some of the most stringent societal restrictions during the COVID-19 pandemic, which may have had a greater impact on the mental health of CYP in the conurbation.^18^ Our analysis by ethnicity was restricted to three groups (white, black and Asian), in part, due lack of statistical power resulting from small numbers in some subgroups. In addition, the broad ‘other’ ethnic group combines a diverse mix of ethnicities limiting the interpretation of the possible impacts of cultural issues on the observed temporal trends. The use of optimal interrupted time series methodology was not possible as the GMCR only became operational in January 2019. Therefore, there was insufficient pre-pandemic data to account for pre-pandemic trends in our models. To enable a comparison with pre-pandemic rates, we divided the 60-month study period into four phases. The cut-off points for each of these phases may be considered subjective. However, this was based on notable temporal changes in societal restrictions and was therefore considered the most pragmatic approach. Finally, as the outcome is relatively rare, there was great variation over time.

### Strengths and limitations in relation to other studies

Our results showed a decrease in self-harm incidence during 2023, following a general increase (for most sociodemographic groups) in pandemic phase 2 of our study. In 2020 and 2021, national secondary school and further education college examinations were cancelled, with affected students receiving their grades based on teachers’ assessments. The normal examination procedures recommenced in the summer of 2022.^19^ A survey carried out by the National Education Union in 2018 found that 82% of educators felt that exams have the greatest impact on the mental health of students and 81% of those in secondary school stated that some students self-harm due to exam-related stress and anxiety.^20^ This survey was conducted prior to the COVID-19 pandemic when national examinations were an expected part of the educational timetable. Therefore, it is reasonable to surmise that the reintroduction of examinations in 2022 had an even greater impact on CYP’s mental health with the initial impact of this reduced in the following year when this once again became normalised. Published hospital data also showed a fall in rates of self-harm during this time. From 2021/2022 to 2022/2023, ED attendances related to self-harm fell by 32% and hospital admissions by 25%.^21 22^ However, these data are reported according to financial year (April-March) and they therefore do not cover the exact same timeframe as our study’s observation period. The Nuffield Trust, which published the hospital admission data (not including ED presentations), suggest that the significant drop in hospital admissions may be due to a change in the way NHS England classifies these data resulting in fewer self-care admissions being recorded in the Hospital Episode Statistics (HES) Admitted Patient Case database.^22^ Although the GMCR data do include retrospective records of hospital attendance, it is primarily based on GP consultations, and therefore this change in recording may have had less of an impact on our results. We considered whether the observed decrease in self-harm rates could be attributed to changes in social media use. A report published by the UK’s Office of Communications (Ofcom) in 2024 concluded that there had not been a discernible reduction in social media usage by CYP in 2023 compared with 2022.^23^ However, results measuring various parameters of parental control of their child’s online activities all showed an increase between these 2 years. For example, in 2023, 69% parents of 16–17-year olds set rules about their child’s mobile phone use compared with 58% in 2022. This may have been a consequence of growing awareness about the risks of harmful content and the resulting UK Government Online Safety Act 2023.^23^ Consistent with other studies self-harm incidence rates among 13–16-year old females were highest throughout our study period.^11^ One of the most striking findings in our results was the increase in rates of self-harm in those aged 10–12 years, particularly in females. In a study examining a similar observation period, an Australian study also found that the highest increase in rates occurred in the youngest females. Between March 2020 and December 2021, those aged 5–14 years increased by 36% compared with 15% in female adolescents aged between 15 and 19.^24^ The authors suggest that factors such as social isolation, internalisation and concerns about their disrupted education may disproportionately impact on female adolescents, particularly during the crucial transition between childhood to adolescence and the associated social, school and biological changes.^24^ The 2024 Ofcom report may also provide some insight into our findings of increased rates in this younger age group. Social media use has increased in younger children with 51% of children under the age of 13 (commonly the minimum age requirement for registering) reporting using social media apps in 2023.^23^ Although the self-harm incidence rate in 17–19-year olds is lower than those aged 13–16 years, analysis of self-harm episodes showed that the rate in 17–19-year-old females was similar to that for 13–16-year-old girls. This would suggest that the older age group may be presenting again with further episodes compared with other age groups. Diggins *et al* found differences in self-harm repetition by age groups with 22–25-year olds most frequently presenting at EDs with multiple episodes.^25^

Other studies examining self-harm rates based on electronic health records have shown a decrease in incidence and prevalence during the early stage of the pandemic. This was possibly due to changes in patients’ access to care with consultation rates reported as decreasing by up to 33%.^26^ Although we also observed a decrease in self-harm incidence rates in males in pandemic phase 1, we found a small increase in rates in females over the same period. It is likely that a greater increase in rates was mitigated by the change in access to GP services. The possible implication of this change in access to healthcare services is illustrated by our secondary analysis of trends in the incidence of anxiety disorders and depression. This showed a significant fall in incidence and prevalence rates, but in comparison, there was no reduction in the rate of antidepressant prescribing. It has been suggested that prescribing data may be a more accurate reflection of the levels of mental distress in the population as it includes the use of a repeat prescription service and is therefore not similarly impacted by any changes in access to primary care.^27^ GMCR data are based on clinical codes submitted into primary care systems, resulting from GP contact or following a hospital visit. Similar to the recorded incidence of anxiety disorders and depression, a self-harm episode will only be included in these analyses if the patient has interacted with healthcare services regarding this behaviour. Therefore, it is not possible to know whether any increase or decrease in rates occurred due to changes in incidence within the population or to differences in help-seeking behaviour or health service delivery and/or clinical recording methodology.^22^

Self-harm incidence rates were highest in CYP of white ethnicity for both males and females. However, in females, our results suggest greater increases (in pandemic phase 2) in black and Asian ethnic groups. This was also observed in a study examining presentations in EDs in the English cities of Derby, Oxford and Manchester. The authors suggest that this may be partly due to cultural differences in help-seeking behaviour and the greater proportion of ethnic minorities residing in more deprived communities.^6^ As is widely reported in the published literature, our results showed that self-harm incidence rates are greatest in the most deprived neighbourhoods.^5^ However, the greatest rate increases (particularly in pandemic phase 2) were observed in the least deprived neighbourhoods. This was also reported by Trafford *et al*,^11^ from a study conducted using nationally representative UK primary care data. The authors found that an overall increase in self-harm rates among females was attributed largely to the especially large rise that occurred in the least deprived quintile. Our results additionally show an increase in incidence in this quintile in males. This comparable study was based on UK-wide electronic health records, rather than solely GM-based, and as such incorporated data with a lower proportion of patients residing in areas of higher social deprivation. A study based on a socioeconomically deprived community in London illustrates how regional and cultural differences may influence results.^28^ Although levels of mental distress were high, incidence of self-harm was low due to influences of community solidarity and a culture of self-reliance. In addition, study participants reported a risk of diminishing social status by being viewed as having mental ill-health and being known to have self-harmed.^28^ Increased self-harm rates in the least deprived communities may reflect greater willingness to seek help, and a disparity in healthcare provision. Results from a study based in three cities in the UK showed that those from the most deprived areas were least likely to present to hospital and, of those that did, they were least likely to be referred on to specialist mental health services.^29^ It is widely reported that the COVID-19 pandemic had a greater impact on individuals and families in lower socioeconomic neighbourhoods, thereby widening pre-existing inequalities.^30^ However, it is not known how the unique set of circumstances with the educational environment in 2022 may have impacted on students from different socioeconomic backgrounds. It is possible that CYP from the least deprived backgrounds experienced heightened levels of mental distress during this time from increased parental pressure to achieve academically.

### Clinical Implications

Results from our study are potentially encouraging, with a decrease in self-harm rates observed during 2023. This also concurs with published hospital admissions and ED data.^21 22^ However, more research is needed to confirm this finding, and to discern the mechanisms driving this trend. Incidence rates among young teenage girls are of particular concern and the rise in self-harm rates observed in 10–12-year olds may suggest younger CYP are increasingly being exposed to risk factors for these harmful behaviours such as increased and detrimental use of social media. Inequalities in health outcomes and access to services are also highlighted and it is essential to identify risks of self-harm and barriers to care for different sectors of society and to enable appropriate and timely care for mental health problems in all CYP.

## Supplementary material

10.1136/bmjment-2025-301615 online supplemental file 1

## Data Availability

Data may be obtained from a third party and are not publicly available.
