# Supplementary material for: Self-harm incidence among children and young people 2019–2023: time series analysis of electronic health records in Greater Manchester, England
Source: BMJ Ment Health. 2025 Jun 9;28(1):e301615. doi: 10.1136/bmjment-2025-301615 (PMC12161433; doi:10.1136/bmjment-2025-301615)

<u>Supplementary Materials</u>

Table S1. Population denominators, numerator values and incidence rates per 100,000 person-months for first recorded self-harm episodes

	Mean population denominator per month N (%)	Total number of first recoded episodes (Jan 2019 to Dec 2023)	Mean number of first recorded self-harm episodes per month	Incidence rate per 100,000 person-months
**Gender**				
Males	300,220 (50.5)	3,767	62.8	21
Females	293,881 (49.5)	9,266	154.4	53
Total	594,101 (100.0)	13,033	217.2	-
**Ethnicity***				
Males				
White	202,388 (67.4)	2,760	46.0	23
Black	19,453 (6.5)	67	1.1	6
Asian	54,171 (18.0)	223	3.7	7
Other	24,209 (8.1)	568	9.5	-
Missing data	0 (0)	149	2.5	-
Females				
White	198,878 (67.7)	6,816	113.6	57
Black	18,842 (6.4)	265	4.4	24
Asian	52,615 (17.9)	608	10.1	19
Other	23,546 (8.0)	1,284	21.4	-
Missing data	0 (0)	293	4.9	-
**Age Group in Years**				
Males				
10-12	63,141 (21.0)	263	4.4	7
13-16	79,195 (26.4)	1,013	16.9	21
17-19	55,583 (18.5)	934	15.6	28
20-24	102,300 (34.1)	1,557	30.0	25
Females				
10-12	60,177 (25.0)	656	10.9	18
13-16	74,890 (25.5)	4,321	72.0	96
17-19	53,230 (18.1)	2,090	34.8	66
20-24	105,583 (35.9)	2,199	36.7	35
**IMD****				
Males				
1	131,140 (43.7)	1,821	30.4	23
2	60,088 (20.0)	877	14.6	24
3	34,590 (11.5)	443	7.4	21
4	35,301 (11.8)	339	5.7	16
5	30,820 (10.3)	278	4.6	15
Missing data	8,280 (2.8)	9	0.2	-
Females				
1	125,963 (42.9)	4,335	72.3	59
2	60,587 (20.6)	2,170	36.2	61
3	34,943 (11.9)	1,099	18.3	54
4	33,871 (11.5)	883	14.7	45
5	29,968 (10.2)	769	12.8	44
Missing data	8,549 (2.9)	10	0.2	-

**Ethnicity population denominator is based on the proportional distribution of Census 2021(24) where there is no missing data*

***Neighbourhood-level Index of Multiple Deprivation (IMD): 1=Most deprived, IMD 5=Least deprived*

Table S2. Temporal trends in rate ratios* (vs. the pre-pandemic period, 95% CI) of incidence episodes of self-harm stratified by sex and age group

	Pandemic phase 1	Pandemic phase 2	Post-Pandemic
**Sex**			
Males	0.90 (0.78-1.04)	0.91 (0.80-1.05)	0.72 (0.62-0.84)
Females	1.07 (0.94-1.22)	1.18 (1.04-1.34)	0.85 (0.74-0.99)
	**Pandemic phase 1**	**Pandemic phase 2**	**Post-Pandemic**
**Age Group**			
**Males**			
10 to 12	0.80 (0.55-1.15)	1.13 (0.81-1.58)	1.08 (0.75-1.56)
13 to 16	0.83 (0.68-1.01)	1.08 (0.90-1.29)	0.88 (0.72-1.08)
17 to 19	1.02 (0.85-1.22)	0.87 (0.73-1.05)	0.67 (0.55-0.83)
20 to 24	0.91 (0.79-1.04)	0,80 (0.70-0.91)	0.61 (0.52-0.71)
**Females**			
10 to 12	1.19 (0.90-1.58)	1.91 (1.47-2.48)	1.60 (1.20-2.14)
13 to 16	1.05 (0.88-1.25)	1.24 (1.05-1.47)	0.87 (0.73-1.05)
17 to 19	1.18 (1.02-1.37)	1.07 (0.93-1.24)	0.77 (0.65-0.90)
20 to 24	0.95 (0.82-1.09)	0.99 (0.86-1.14)	0.72 (0.61-0.84)

**Rate ratios are compared to the pre-pandemic period as the generic reference category (set as 1.00).*

Table S3. Temporal trends in rate ratios* (vs. the pre-pandemic period, 95% CI) of incidence episodes of self-harm stratified by ethnicity and neighbourhood deprivation quintile

	Pandemic phase 1	Pandemic phase 2	Post-Pandemic
**Ethnicity**			
**Males**			
White	0.97 (0.86-1.08)	0.91 (0.81-1.01)	0.73 (0.64-0.82)
Black	1.06 (0.52-2.16)	1.08 (0.55-2.11)	0.96 (0.46-2.03)
Asian	0.69 (0.47-1.02)	0.90 (0.63-1.28)	0.80 (0.54-1.18)
**Females**			
White	1.05 (0.92-1.20)	1.15 (1.01-1.30)	0.86 (0.74-0.99)
Black	1.00 (0.68-1.46)	1.53 (1.09-2.16)	1.19 (0.81-1.74)
Asian	0.96 (0.76-1.22)	1.32 (1.06-1.65)	0.82 (0.63-1.06)
**IMD****			
**Males**			
IMD1	0.84 (0.72-0.97)	0.82 (0.71-0.95)	0.72 (0.61-0.85)
IMD2	1.06 (0.88-1.27)	0.94 (0.78-1.13)	0.77 (0.62-0.95)
IMD3	0.97 (0.75-1.26)	0.99 (0.77-1.27)	0.51 (0.37-0.71)
IMD4	0.86 (0.64-1.16)	0.95 (0.71-1.26)	0.64 (0.46-0.90)
IMD5	1.05 (0.73-1.52)	1.42 (1.01-1.98)	1.25 (0.87-1.81)
**Females**			
IMD1	0.97 (0.85-1.11)	1.15 (1.01-1.31)	0.84 (0.73-0.98)
IMD2	1.11 (0.96-1.28)	1.08 (0.93-1.24)	0.82 (0.70-0.97)
IMD3	1.18 (1.00-1.41)	1.24 (1.04-1.46)	0.83 (0.68-1.01)
IMD4	1.11 (0.89-1.39)	1.24 (1.00-1.54)	0.87 (0.68-1.11)
IMD5	1.32 (1.03-1.69)	1.54 (1.21-1.95)	1.07 (0.82-1.40)

**Rate ratios are compared to the pre-pandemic period as the generic reference category (set as 1.00).*

***Neighbourhood-level Index of Multiple Deprivation (IMD): 1=Most deprived, IMD 5=Least deprived*

Figure S1: Temporal trends in self-harm episodes by sex. a) Episode rates per 100,000 person-months (95% CI) and b) incidence rate ratios (IRR) by sex (vs. the pre-pandemic period, 95% CI).

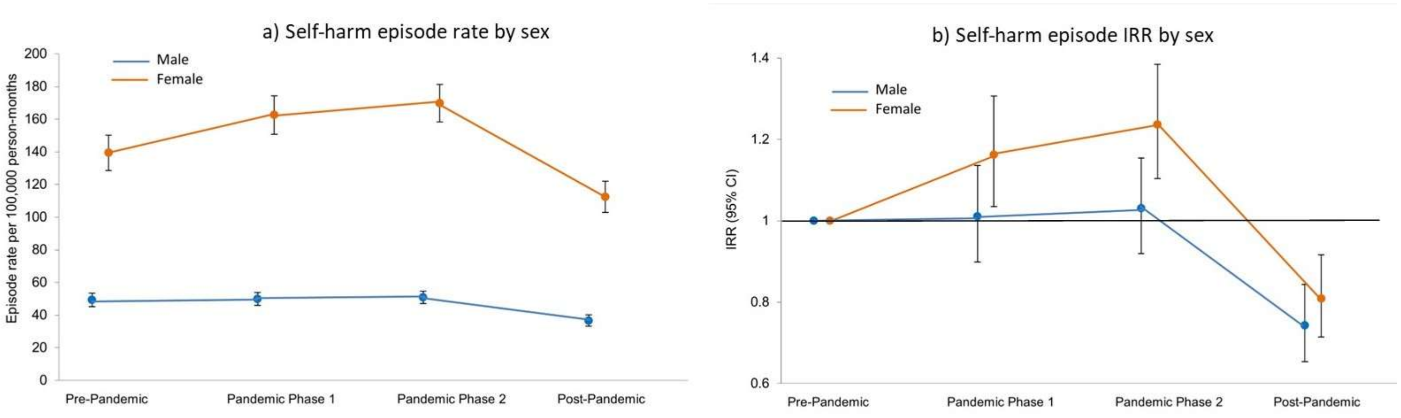

Figure S2: Temporal trends in self-harm episodes by age group. a and c) Episode rates per 100,000 person-months (95% CI) and b and d) incidence rate ratios (IRR) by sex (vs. the pre-pandemic period, 95% CI).

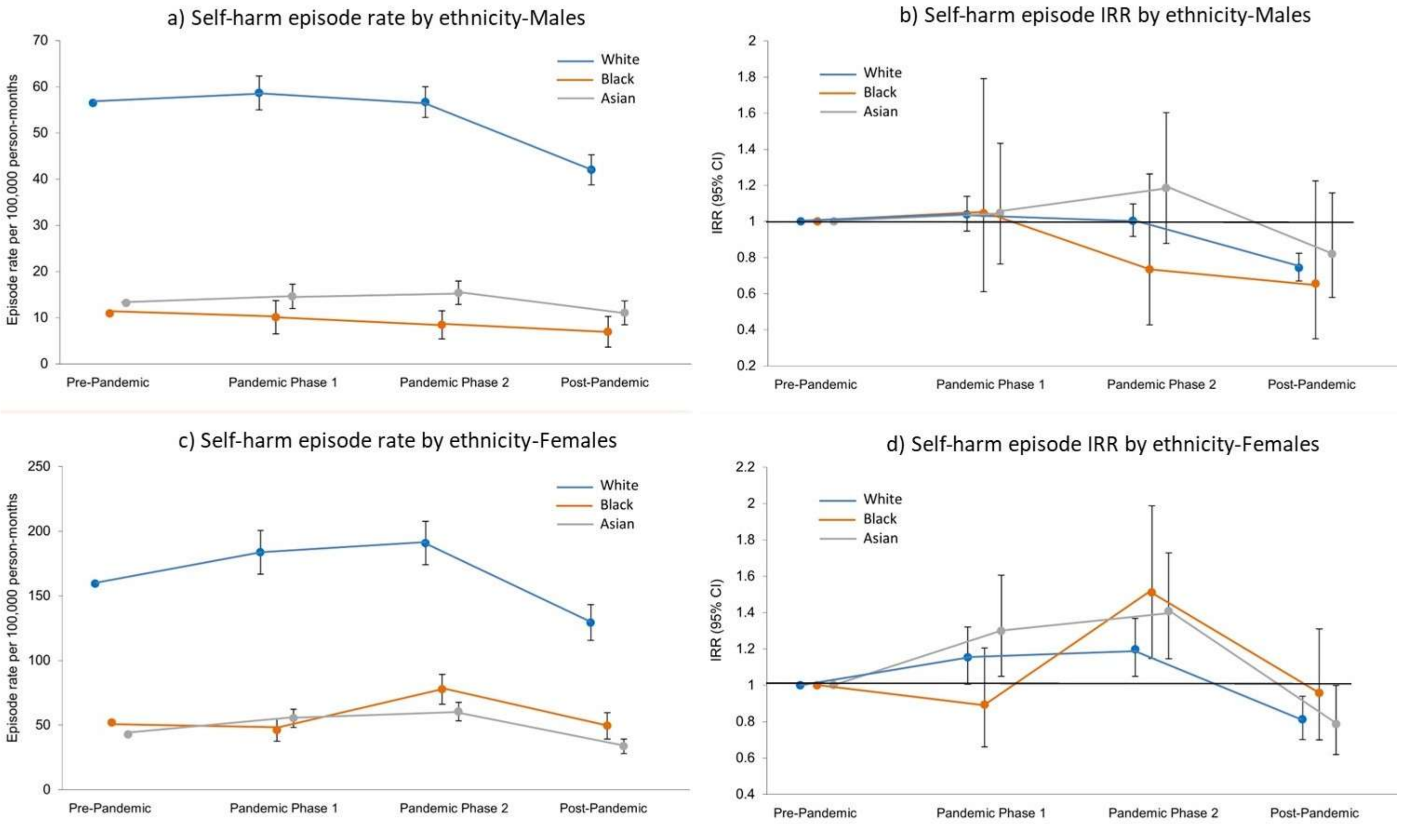

Figure S3: Temporal trends in self-harm episodes by ethnicity. a and c) Episode rates per 100,000 person-months (95% CI) and b and d) incidence rate ratios (IRR) by sex (vs. the pre-pandemic period, 95% CI).

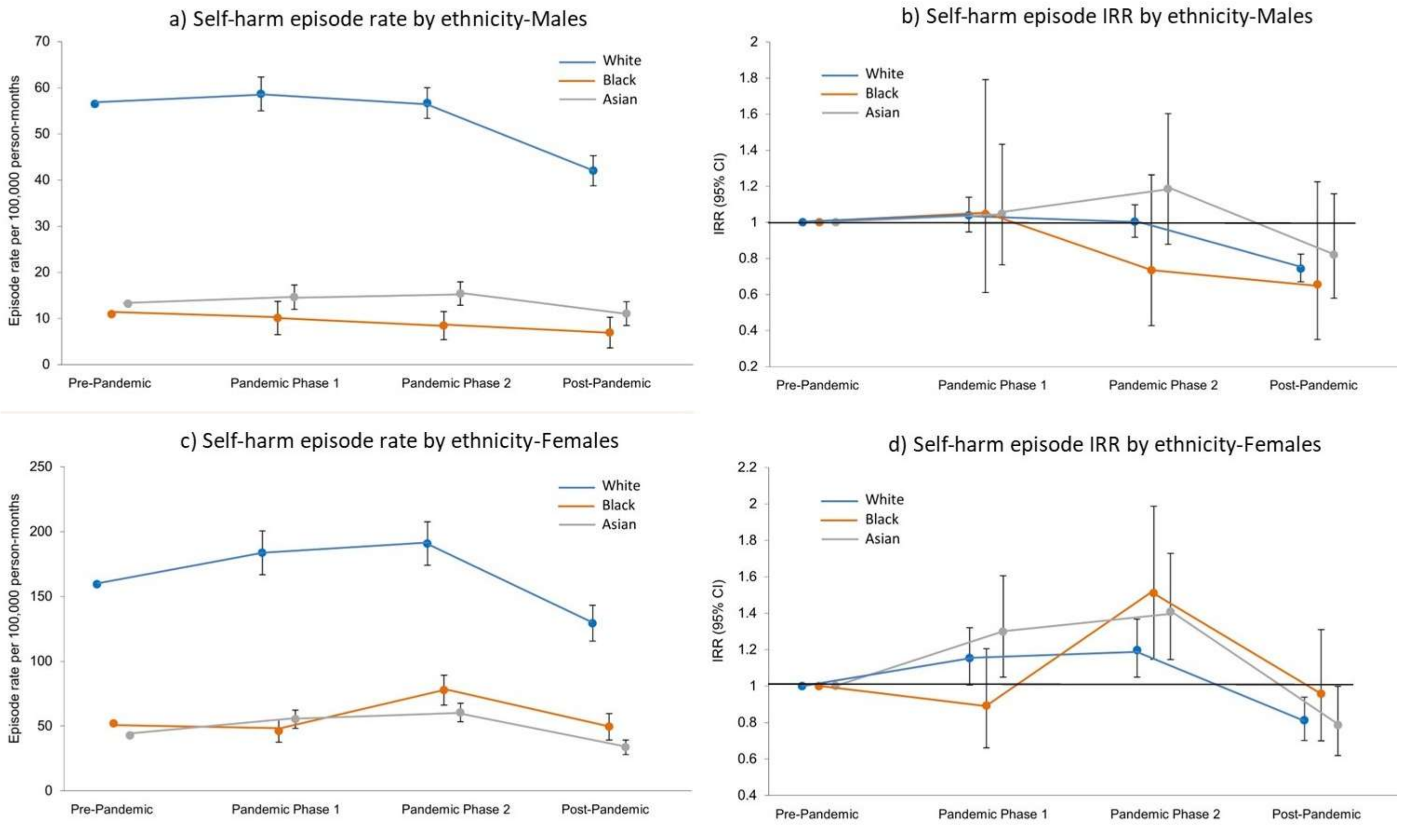

Figure S4: Temporal trends in self-harm episodes by neighbourhood deprivation quintile*. a and c) Episode rates per 100,000 person-months (95% CI) and b and d) incidence rate ratios (IRR) by sex (vs. the pre-pandemic period, 95% CI).

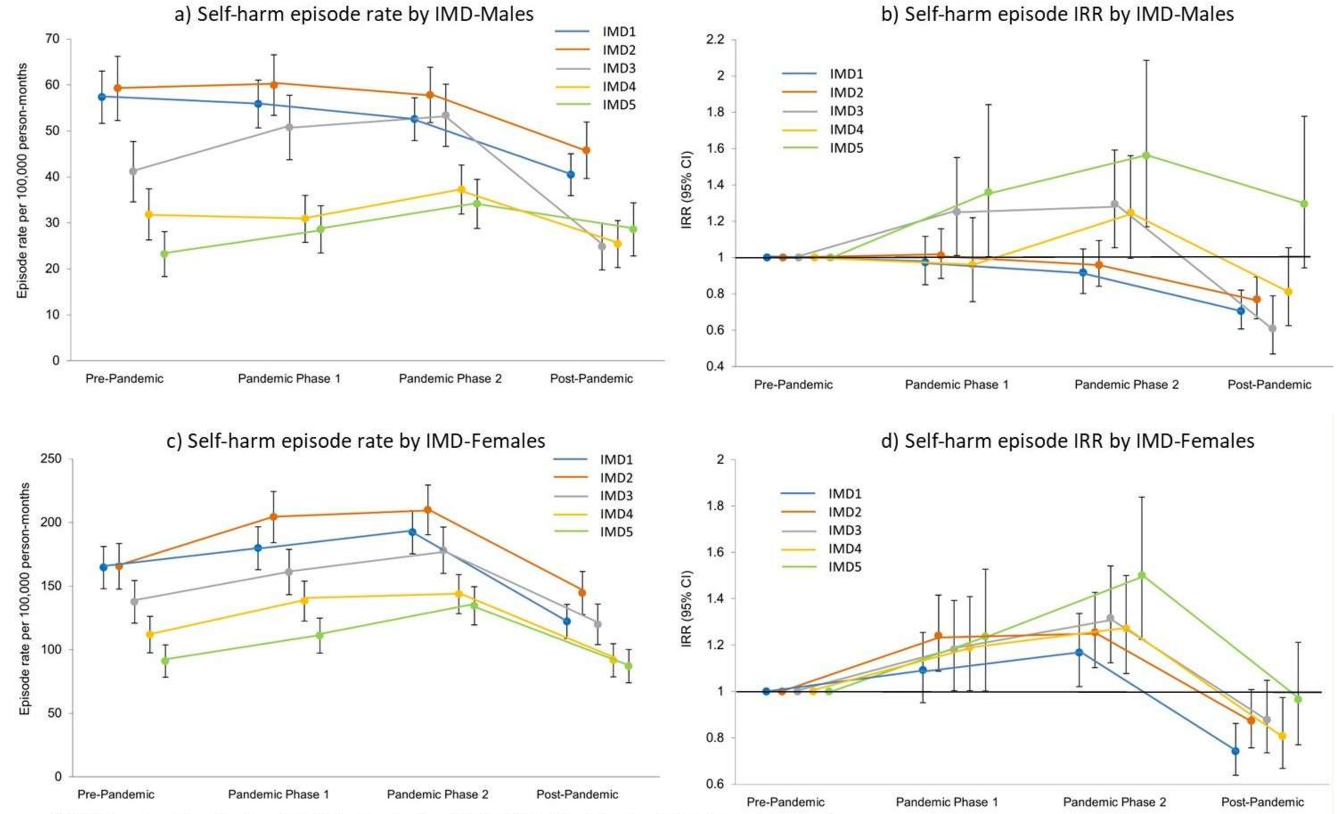

**Neighbourhood-level Index of Multiple Deprivation (IMD): IMD1=Most deprived, IMD 5=Least deprived*

Figure S5: Temporal trends in anxiety disorders and depression incidence by sex. a) Incidence rates per 100,000 person-months (95% CI) and b) incidence rate ratios (IRR) by sex (vs. the pre-pandemic period, 95% CI).

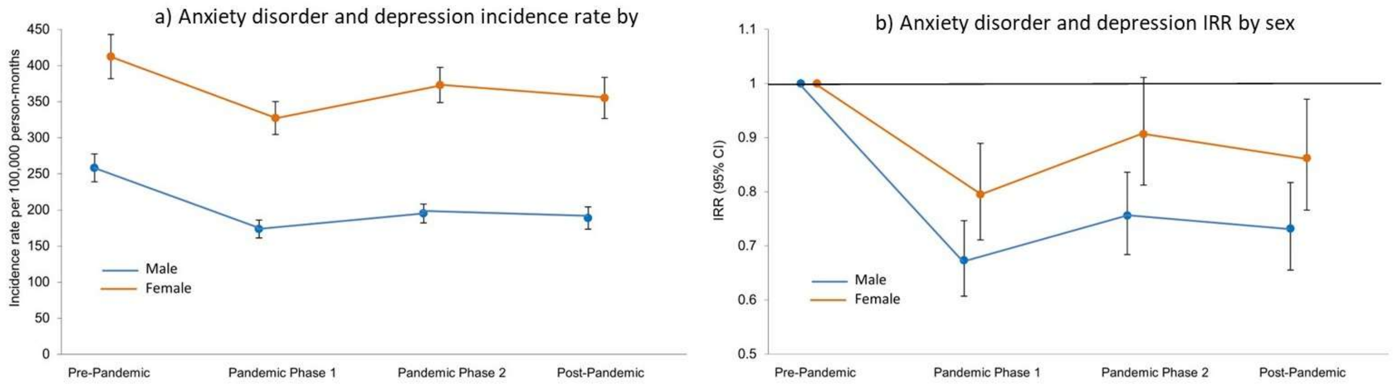

Figure S6: Temporal trends in anxiety disorders and depression episodes by sex. a) Episode rates per 100,000 person-months (95% CI) and b) incidence rate ratios (IRR) by sex (vs. the pre-pandemic period, 95% CI).

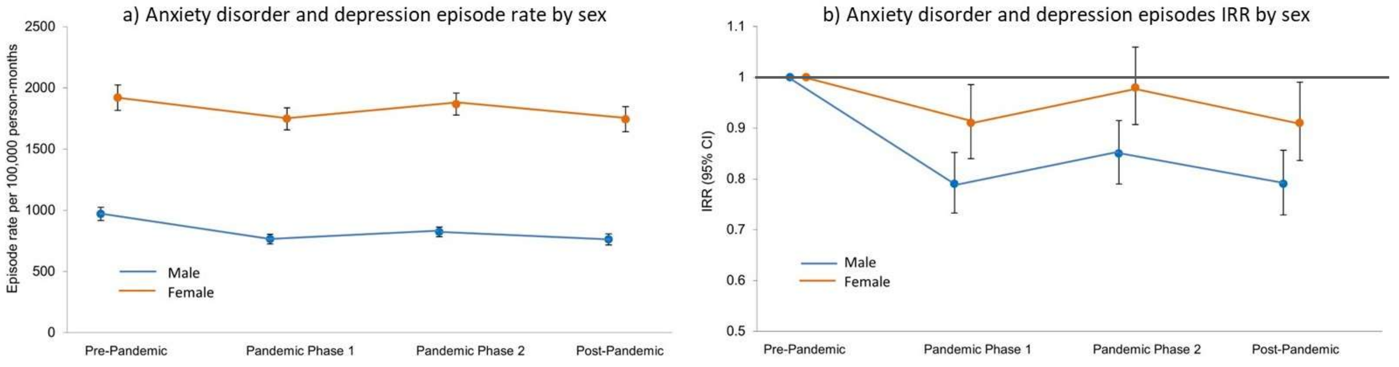

Figure S7: Temporal trends in antidepressant prescribing by sex. a) Prescribing rates per 100,000 person-months (95% CI) and b) incidence rate ratios (IRR) by sex (vs. the pre-pandemic period, 95% CI).

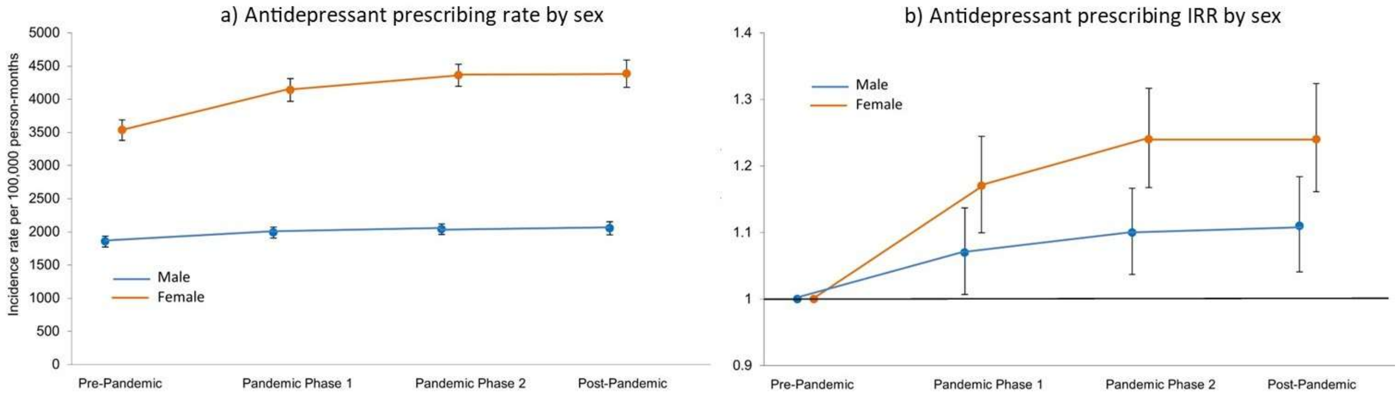

Supplement: online supplemental file 1 [file bmjment-28-1-s001.pdf]
